# No relationship between foot posture and frontal knee alignment in healthy adolescents

**DOI:** 10.1186/1757-1146-7-S1-A56

**Published:** 2014-04-08

**Authors:** Shinsuke Matsumoto, Shigeharu Tanaka

**Affiliations:** 1 Dept. of Physical Therapy, Kawasaki Junior College of Rehabilitation, Kurashiki, Okayama, 701-0192, Japan

## Background

Foot posture has been suggested to be related to the development of lower-limb musculoskeletal conditions because of its potential influence on the mechanical alignment and dynamic function of the lower limb. During most weight bearing activities, the posture and motion of the foot and knee are coupled within a closed kinematic chain. The exact relationship, however, between them in healthy individuals is not known. The purpose of this study was therefore to investigate if foot posture was related with frontal knee alignment in healthy adolescents.

## Methods

The foot posture and frontal knee alignment of Forty-eight healthy individuals ( 27 females, average age 21.1±2.8 yr, BMI 21.0±1.9 ) was assessed and then analyzed to determine if any relationship exist between them.

The foot posture measurement was evaluated using FPI [1]. FPI values ranged from -2 to +2 for each of the six criteria and from -12(highly supinated) to +12(highly pronated) for the total score. The raw FPI scores were converted to transformed scores to allow the scores to be used as interval data for statistical analysis.

The Knee alignment measure was performed by measuring the femoral tibial angle (FTA) with a goniometer [2]. The axis of the goniometer was positioned over the centre of the patella and the arms were aligned with the mid-thigh and with the tibial shaft.

Means of the FPI score and FTA were compared by gender using Student's *t*-test. Pearson’s correlation coefficient was used to investigate the relationship between the FPI score and FTA.

## Results

There was no difference between FTA of males and females (176.5 vs. 176.7; p=0.792). The significant difference in FPI score between males and females was found (5.95 vs. 2.85; p=0.001). No relationship was found between the FPI score and FTA (r = 0.006, p = 0.978).

## Conclusion

Static foot posture as quantified by FPI and frontal knee alignment as quantified by FTA do not seem to correlate each other in healthy adolescents. These results should be interpreted with caution due to a small sample size.
